# Are naringenin and quercetin useful chemicals in pest-management strategies?

**DOI:** 10.1007/s10340-013-0535-5

**Published:** 2013-11-19

**Authors:** Sylwia Goławska, Iwona Sprawka, Iwona Łukasik, Artur Goławski

**Affiliations:** 1 Department of Biochemistry and Molecular Biology, Siedlce University of Natural Sciences and Humanities, Prusa 12, 08-110 Siedlce, Poland; 2 Department of Zoology, Siedlce University of Natural Sciences and Humanities, Prusa 12, 08-110 Siedlce, Poland

**Keywords:** *Acyrthosiphon pisum*, EPG, Artificial diet, Sucrose–agarose gel, Antifeedants, Naringenin, Quercetin

## Abstract

The effects of two polyphenolic flavonoids (flavanone naringenin and flavonol quercetin) on development, fecundity, and mortality of the pea aphid, *Acyrthosiphon pisum* Harris (Hemiptera: Aphididae), were determined in vitro, on an artificial diets. Also determined in vitro (DC EPG method), on sucrose–agarose gels, were the effects of flavonoids on the probing and feeding behavior of adult apterae. When added to a liquid diet, higher concentrations of studied flavonoids increased the developmental time, the pre-reproductive period, and mortality and decreased fecundity and the intrinsic rate of natural increase of *A. pisum*. In most events associated with stylet activity (as indicated by EPG waveform g-C), differences in probing behavior did not statistically differ between the control gel and those with flavonoids; quercetin at 10, 100, and 1,000 µg cm^−3^ prolonged the number of gel penetrations; and quercetin only at 10,000 μg cm^−3^ prolonged the time the first g-C waveform was observed. Addition of flavonoids to the gels generally reduced passive ingestion from fluids of the gels (EPG waveform g-E2). At higher concentrations (>1,000 µg cm^−3^) the flavonoids completely stopped salivation (EPG waveform g-E1) and passive ingestion from fluids of the gels (EPG waveform g-E2). In events associated with active ingestion (EPG waveform g-G), however, differences in feeding behavior did not statistically differ between the control gel and those with flavonoids. The present findings demonstrate detrimental effects of the flavanone naringenin and flavonol on the behavior of the pea aphid. This can be employed in a biotechnological projects for plant breeding resistant to herbivores, including aphids.

## Introduction

Aphids have traditionally been controlled by application of insecticides, but chemical control has many disadvantages (Blackman and Eastop 2000; Despres et al. 2007; Rharrabe et al. 2007). Due to the known harmful effects of pesticides, there is a growing use of alternative strategies of insect control, including the use of resistant cultivars, transgenic plants containing novel genes that confer resistance to phloem-feeding insects, and chemicals that show bioactivity against insects (Horowitz and Ishaaya 2004; Rharrabe et al. 2007). Understanding whether and how these alternative methods affect aphid behavior are crucial to integrated pest management.

Plants synthesize a wide array of secondary metabolism compounds that are generally thought to be involved in plant–insect interactions (Kubo 2006). Such compounds, like flavonoids, confer some resistance against herbivores (Scalbert 1991; Marles et al. 2003; Simmonds 2003; Dixon et al. 2005; Goławska et al. 2006, 2008; Goławska and Łukasik 2009). They can act as repellent, antifeedant, or toxic (Wollenweber 1994; Rao 1982). They may promote oxidative stress within insect tissues (Łukasik et al. 2009, 2011). Flavonoids constitute an interesting family synthesized and accumulated by plants (Felgines et al. 2000). The number of flavonoids, their structural diversity, and bioactivity make these compounds one of the most important group of natural origin substances (Harborne 1988). Flavonoids are plant secondary metabolites resulting from the addition of malonyl CoA to the phenylpropanoid molecule coumaroyl CoA (Winkel-Shirley 2002; Lepiniec et al. 2006). These polyphenolic compounds are characterized by two aromatic cycles linked by a heterocycle. They are classified according to the oxidation degree of the C-ring, and include flavonols, anthocyanins, and flavan-3-ols. These molecules can undergo modifications of their aromatic cycles, including hydroxylations, methylations, glycosylations, acylations, or prenylations, which account for the diversity within a compound class (Winkel-Shirley 2006). The most important physiological functions of flavonoids are their protection of photosynthetic system and DNA from UV-B radiation damage and attacks by fungi and animals (Harborne and Williams 2000; Morimoto et al. 2000; Rharrabe et al. 2007; Widstrom and Snook 2001). Flavonoids have been exploited for their medicinal and nutritional activities (Erdman et al. 2007; Mink et al. 2007). Interest of nutritional importance of plant flavonoids arose mostly due to their antioxidant activity (Halaweish et al. 2003). They became of interest both human and animal nutrition. Flavonoids have also been shown to possess anti-inflammatory, antiallergenic, antimicrobial, estrogenic, and pharmacological activities in mammals (Miksicek 1993; Rice-Evans et al. 1996; Benavente-Garcia et al. 1997; Di Carlo et al. 1999; Dixon and Steele 1999).

Advances in the knowledge about the health benefits of flavonoids, in both crop and medicinal plants, have prompted plant breeders to look for increase levels of these compounds in crops (Johnson and Felton 2001; Galili et al. 2002). Flavonoids also could be useful in a pest-management strategy involving transgenic plants that express specific flavonoids. Such genetic engineering has been demonstrated (Yu et al. 2003; Deavours and Dixon 2005). Therefore, metabolic engineering of flavonoids, to enhance the dietary intake of these compounds has aroused great interest (McCue and Shetty 2004; Deavours and Dixon 2005). Although there has been some research on the chemical and physiological effects of flavonoids on insects, their precise mode of insecticidal action is not fully understood. Moreover, there has been very little research on the influence of increasing the levels of specific flavonoids in plants on the behavior of insect, especially feeding behavior. Moreover, if pest-management strategies are to be based on the use of transgenic crops that express specific flavonoids, more information on flavonoids modes of activity will be required to ensure predictable and durable control in the field. While flavonoids can have insecticidal activity, they could also benefit insect pests and make them more difficult to control with biological control agents, such as viral pathogens (Simmonds 2001, 2003).

In the present research, the effects of two flavonoids compounds from different classes, flavanone naringenin and flavonol quercetin, on *Acyrthosiphon pisum* (Hemiptera, Aphididae), were studied in in vitro. The effects of these flavonoids on development, fecundity, and mortality of the pea aphid were studied using an artificial diet supplemented with studied flavonoids and the electrical penetration graph (EPG) method was used to monitor the feeding behavior of pea aphids exposed to the studied flavonoids in an sucrose–agarose gel. No previous study has examined the effects of these flavonoids on the development and feeding behavior of the pea aphid. Understanding the activity of these compounds should (1) clarify the appropriateness of using the candidate genes for a given agronomical purpose; (2) help researches overcome the difficulties in using flavonoids in engineering strategies to construct genetically modified plants that resist insects (Sauvion et al. 2004).

## Materials and methods

### Aphid culture

Females of the pea aphid, *A. pisum* Harris, were obtained from a stock culture kept at the Siedlce University of Natural Sciences and Humanities, Poland. The stock culture was maintained on broad bean (*Vicia faba* L. var. Start (Fabaceae)) in plastic pots in an environmental chamber at 21 ± 1 °C, L16:D8 photoperiod, and 70 % RH. Before the experiments, females of *A. pisum* were maintained in the laboratory for duration of one full generation period (Apablaza and Robinson 1967). Adult apterous females were used for all experiments. Aphid cohort production was as described earlier (Rahbe and Febvay 1993).

### Chemicals

Naringenin and quercetin were purchased from Sigma-Aldrich (CN. 67604482, CN. 117395, respectively). All other dietary components were obtained from Sigma (Sigma Chemical Co., Poznań, Poland).

### Aphid bioassays on artificial diets

The tests were conducted in an environmental chamber at 21 ± 1 °C, L16:D8 photoperiod, and 70 % RH. Liquid artificial diet containing an approximately optimal composition of essential nutrients (Ackey and Beck 1972) was used for the oral delivery of studied flavonoids to *A. pisum*. Flavonoids, naringenin and quercetin, were incorporated into diet at five concentrations 1, 10, 100, 1,000, and 10,000 µg cm^−3^. Control diets (without flavonoids) were also included. After components had dissolved, the diets were sterilized by filtration through 0.45-µm Millipore filters. A total of 0.5 cm^3^ of solution was added to each aphid feeding chamber, which consisted of plastic rings (35 mm diameter, 15 mm height) overlain with two layers of stretched Parafilm M^®^; the diet was placed between the two layers of Parafilm. For assays *A. pisum* were placed on control diet (without flavonoids) and left to produce nymphs overnight. Adults were then removed, and the nymphs were maintained on the diet for a further 24 h. Then group of five nymphs were placed on the each feeding chamber in the presence of 1, 10, 100, 1,000, and 10,000 µg cm^−3^ of naringenin, quercetin, or control diet. Ten replicates were done for control and each flavonoids concentration.

Larval developmental time (pre-reproductive period), daily fecundity, and mortality of the aphids were monitored daily for 15 days. Feeding sachets were replaced every 2 days to avoid contamination and deterioration of the diet. The collected data were used to calculate the average time of generation development (*T*) and the intrinsic rate of natural increase (*r*_m_) according to the equations of Wyatt and White (1977):

$$\begin{aligned} T & = d/0.74 \\ r_{\text{m}} & = [0.74(\ln {\text{Md}})]/d, \\ \end{aligned}$$

where *d* is the length of the pre-reproductive period, Md is the number of larvae born in the reproduction period which equals the *d* period, and 0.74 is the correction factor.

### Aphid feeding behavior

The effect of flavonoids on pea aphid feeding behavior was investigated in vitro, using sucrose–agarose gels. Gels were prepared by incorporating 1.25 % agarose (Sigma A-0169) into a 30 % sucrose solution and then adding one of the flavonoids to obtain concentrations of 0 (control), 1, 10, 100, 1,000, and 10,000 µg cm^−3^. After the mixtures were stirred, they were heated in a water bath (75 °C for 30 min) and then poured into plastic rings (10 mm high and 15 mm diameter) covered with a stretched Parafilm M^®^ membrane. Transparent gels formed after 1–2 min and were offered to aphids for probing.

EPGs (Tjallingii 1988) were used to monitor the probing and feeding behavior of the adult aphids that were exposed to flavonoids in an gel. The electrical penetration graph technique (EPG) makes it possible to record different waveform patterns related to aphid activities and stylet locations during penetration, usually within the plant tissue (Sauvion and Rahbe 1999). Apterous adults were collected between 6 and 7 a.m., then dorsally tethered on the abdomen with a gold wire (2 cm long, 20 µm in diameter) and water-based conductive silver paint (Demetron, L2027, Darmstadt, Germany). After the aphids were starved for 2 h, they were carefully transferred to the gels. The tethered aphids were individually placed on the surface at the center of each gel, and a second electrode was introduced into the gel. Aphids were connected to a Giga-4 EPG amplifier (Wageningen, Agricultural University, Entomology Department, The Netherlands) coupled to a IBM compatible computer through a DAS 8 SCSI acquisition card (Keithley, USA). During EPG recordings, aphids were in a Faraday cage in the laboratory (21 ± 1 °C, L16:D8 photoperiod, and 70 % RH). EPG recordings began between 9 and 10 a.m. on both control and flavonoid gels. EPG recordings were made for ten aphids on gels without flavonoids (control) and for ten aphids for each flavonoid (naringenin, quercetin) concentration (1, 10, 100, 1,000, and 10,000). Aphid feeding behavior was monitored for 2 h.

EPGs were acquired and analyzed with STYLET 2.2 software provided by W.F. Tjallingii. Waveforms were identified according to waveforms found by others in artificial diets and sucrose–agarose gels (Sauvion and Rahbe 1999; Sauvion et al. 2004; Goławska 2007; Cid and Fereres 2010; Sprawka and Goławska 2010) as indicative of different feeding activities according to Tjallingii (1990) and called g-np, g-C, g-E1, g-E2, and g-G. In this study, the probing and feeding behavior on gels we used the same nomenclature with the prefix g to describe the feeding waveforms based on the first letter of the name gel (g—gel sucrose–agarose). The main waveforms generated by aphids probing and feeding on gels are interpreted to be analogous to or representative of the waveforms previously described for the probing and feeding of aphids on plants (Tjallingii 1988, 1990, 1994). In waveform g-np, the aphids stylet is outside the gel (analogous to the stylet being outside the plant, pattern np on plants). Waveform g-C indicates stylet activity in the gel (analogous to the stylet penetrating the epidermis and masophyll, before salivation and ingestion, pattern C on plants). Waveform g-E1 indicates salivation into the gel (analogous to the stylet salivating into phloem sieve tubes, pattern E1 on plants). Waveform g-E2 indicates passive ingestion of fluids from the gel (analogous to the stylet passively ingesting phloem sap, pattern E2 on plants). Waveform g-G indicates active ingestion of the fluids from the gel (analogous to the stylet actively ingesting xylem sap, pattern G on plants). EPG parameters were measured in each group and recalculated per one insect.

### Statistical analysis

The differences in pea aphid population development were carried out by a factorial analysis of variance (ANOVA) followed by the post hoc Fisher’s LSD test. The factorial ANOVA included an assessment of the artificial diet effect (two experimental factors: (1) compound (naringenin, quercetin); (2) concentration (0, 1, 10, 100, 1,000, and 10,000 µg cm^−3^) on pea aphid population parameters (the pre-reproductive period, mean daily fecundity, the intrinsic rate of natural increase, the average time of generation development, and mortality). The values of the EPG parameters (the duration of stylet activity in the gel, duration of salivation into the gel, duration of passive and active ingestion from the gel, and the number of penetrations) were analyzed with the Kruskal–Wallis test followed by the post hoc multiple comparisons of mean ranks for all groups. All analyses used Statistica for Windows version 9.0 (Statsoft 2011).

## Results

### *Acyrthosiphon pisum* performance on diets with naringenin and quercetin

The statistical analysis showed an effect of dose. The tested concentrations statistically affected the pre-reproductive period (Factorial ANOVA; *F*_11,48_ = 5.64; *p* < 0.001), fecundity (Factorial ANOVA; *F*_11,48_ = 20.07; *p* < 0.001), *r*_m_ (Factorial ANOVA; *F*_11,48_ = 16.33; *p* < 0.001), *T* (Factorial ANOVA; *F*_11,48_ = 6.05; *p* < 0.001), and mortality (factorial ANOVA; *F*_11,48_ = 89.37; *p* < 0.001) (Table 1). There are no effects of the compound (naringenin and quercetin) on pre-reproduction period, daily fecundity, intrinsic rate of natural increase (*r*_m_), and average time of generation development (*T*). There is only an effect of the compound on the mortality (Table 1). The analysis showed an effect of increasing the concentration of the compounds. Higher concentrations of tested flavonoids increased the pre-reproductive period, decreased fecundity, and increased mortality of adult *apterae* (Table 2). Higher concentrations of tested compounds in the diet also increased *T* and reduced *r*_m_ (Table 2).

**Table 1:** Statistical results of the factorial ANOVA of pea aphid development on a liquid artificial diet as affected by naringenin and quercetin

Parameter	df	*F*	*p*
Pre-reproductive period			
Compound	1	2.880	0.096
Concentration	5	11.650	<0.001
Compound × concentration	5	0.190	0.964
Mean daily fecundity			
Compound	1	0.350	0.557
Concentration	5	43.990	<0.001
Compound × concentration	5	0.090	0.993
*R*_m_			
Compound	1	0.364	0.549
Concentration	5	35.273	<0.001
Compound × concentration	5	0.582	0.714
*T*			
Compound	1	2.460	0.123
Concentration	5	12.590	<0.001
Compound × concentration	5	0.250	0.939
Mortality			
Compound	1	5.434	0.024
Concentration	5	194.898	<0.001
Compound × concentration	5	0.634	0.675

**Table 2:** Population parameters of *A. pisum* as affected by concentration of naringenin and quercetin in the diet (values are mean ± SD)

Parameter	Control	Naringenin and quercetin (μg cm^−3^)				
		1	10	100	1,000	10,000
Pre-reproductive period (days)	9.00 ± 0.00c	9.00 ± 0.47c	9.30 ± 0.48bc	9.50 ± 0.53b	9.70 ± 0.48b	10.30 ± 0.48a
Daily fecundity per female	1.59 ± 0.07a	1.57 ± 0.05a	1.57 ± 0.09a	1.59 ± 0.07a	1.33 ± 0.03b	1.32 ± 0.01b
Mortality	0.30 ± 0.48e	0.50 ± 0.53e	1.80 ± 0.63d	3.30 ± 0.82c	5.50 ± 0.53b	7.70 ± 0.95a
Intrinsic rate of natural increase (*r*_m_)	0.04 ± 0.00a	0.04 ± 0.00a	0.04 ± 0.00a	0.04 ± 0.00a	0.02 ± 0.00b	0.02 ± 0.00b
Average time of generation development (*T*)	12.16 ± 0.00c	12.38 ± 0.48c	12.57 ± 0.65bc	12.84 ± 0.71bc	13.11 ± 0.65b	13.92 ± 0.65a

Mean in row followed by* different letters* are different at *p* < 0.05 (Fisher’s LSD test)

### *Acyrthosiphon pisum* feeding behavior on gels with naringenin and quercetin

EPG recordings indicated that the addition of the flavonoids naringenin and quercetin to the sucrose–agarose gel clearly affected the probing and feeding behavior of *A. pisum*. The naringenin and quercetin statistically affected stylet activity in the gel (g-C waveform), salivation into the gel (g-E1 waveform), passive ingestion from the gel (g-E2 waveform), active ingestion from the gel (g-E2 waveform), and number of penetrations (Kruskal–Wallis test; *p* < 0.001 in all cases). The effect depended on flavonoid concentration (Tables 3, 4). Although aphids probed the gel in the controls and in all flavonoid treatments (as indicated by the presence of g-C waveform), aphids did not exhibit salivation and passive ingestion (as indicated by the absence of g-E1 and g-E2 waveforms) with gels containing ≥10,000 µg cm^−3^ or ≥1,000 µg cm^−3^ of naringenin or quercetin, respectively. All EPG waveforms were observed on gels that contained ≤100 µg cm^−3^ of naringenin or quercetin (Tables 3, 4).

**Table 3:** *Acyrthosiphon pisum* stylet activity (g-C waveform) on an sucrose–agarose gel as affected by naringenin and quercetin

Flavonoid added	Concentration (μg cm^−3^)	Number of penetrations/2 h	Time of the first probing (min)	Average time of probing (min)
Control	0	3.20 ± 1.47b	7.23 ± 5.81b	8.88 ± 5.86b
Naringenin	1	2.90 ± 0.22b	4.81 ± 8.52b	21.95 ± 1.24ac
	10	5.00 ± 0.67ab	18.69 ± 8.49ab	19.85 ± 6.74ab
	100	4.90 ± 0.98ab	17.16 ± 6.74ab	19.51 ± 4.31ab
	1,000	5.30 ± 1.57ab	27.52 ± 8.06ab	29.08 ± 8.16ab
	10,000	4.60 ± 1.39ab	24.53 ± 7.36ab	23.67 ± 6.69ab
Quercetin	1	4.30 ± 1.12ab	25.25 ± 8.91ab	24.73 ± 10.69ac
	10	10.50 ± 1.69a	6.60 ± 2.31ab	5.41 ± 2.03bc
	100	12.10 ± 1.95a	8.14 ± 5.12ab	8.07 ± 1.52ab
	1,000	11.2 ± 2.24a	3.31 ± 1.16ab	3.77 ± 0.59bc
	10,000	4.00 ± 0.76ab	46.14 ± 6.94a	37.79 ± 6.70a

Values were derived from 2-h EPG recordings and are mean ± SE; *n* = 10. Mean in columns followed by* different letters* are different at *p* < 0.05 (Kruskal–Wallis test)

**Table 4:** *Acyrthosiphon pisum* salivation (waveform g-E1), passive ingestion (waveform g-E2), and active ingestion (waveform g-G) on an sucrose–agarose gel as affected by naringenin and quercetin

Flavonoid added	Concentration (μg cm^−3^)	Total time of g-E1 pattern (min)	Total time of g-E2 pattern (min)	Total time of g-G pattern (min)
Control	0	18.74 ± 6.50ab	60.17 ± 8.36a	22.87 ± 10.28abc
Naringenin	1	26.28 ± 4.56a	29.43 ± 3.20a	3.44 ± 7.69ac
	10	37.32 ± 8.82a	17.75 ± 6.42ac	0.00 ± 0.00c
	100	31.15 ± 6.83a	8.31 ± 1.71ac	3.10 ± 2.17ac
	1,000	11.62 ± 4.94ac	0.00 ± 0.00c	32.61 ± 7.68abc
	10,000	0.00 ± 0.00bc	0.00 ± 0.00c	54.59 ± 10.52b
Quercetin	1	12.18 ± 2.21ac	17.89 ± 5.64ac	10.83 ± 5.86abc
	10	36.20 ± 7.71a	29.25 ± 7.98ab	11.28 ± 10.03abc
	100	31.98 ± 7.93a	0.23 ± 0.18bc	12.98 ± 8.58abc
	1,000	22.56 ± 4.72a	0.00 ± 0.00c	54.73 ± 12.46ab
	10,000	0.00 ± 0.00bc	0.00 ± 0.00c	10.29 ± 5.02abc

Values were derived from 2-h EPG recordings and are mean ± SE; *n* = 10. Mean in columns followed by* different letters* are different at *p* < 0.05 (Kruskal–Wallis test)

Although the g-C waveform was exhibited on all gels and although the number of penetrations was affected by the treatments with quercetin at 10, 100, and 1,000 µg cm^−3^ (Table 3), the timing of the first probe was prolonged by quercetin at 10,000 µg cm^−3^ and tended to be prolonged, but without statistical significance, by the higher concentrations of naringenin (Table 3). The average time of probing also tended to be higher with addition of the flavonoids, but statistical differences were observed for naringenin and for quercetin at 1 and 10,000 µg cm^−3^ (Table 3).

The higher concentrations of naringenin and quercetin (1,000 and 10,000 μg cm^−3^) reduced or completely inhibited aphid salivation (waveform g-E1) and passive ingestion (waveform g-E2) (Table 4). Both flavonoids at 10,000 μg cm^−3^ reduced to zero the total time that pea aphids salivated into the gels, but the effect was not statistically significant due to the high variability of the data (Table 4). A similar tendency was observed for passive ingestion from gels for both flavonoids at 1,000 and 10,000 µg cm^−3^ (Table 4). Quercetin at 100 μg cm^−3^ reduced the duration of passive ingestion up to 260 times, and no passive ingestion occurred with naringenin or quercetin at 1,000 and 10,000 μg cm^−3^. This phase of aphid feeding was generally reduced at the lower concentrations of both flavonoids and was completely stopped by the higher concentrations (Table 4).

As indicated by the g-G waveform, the flavonoids tended to delay, prolong, or inhibit active ingestion, but the effect was not statistically significant (Table 4).

## Discussion

Our study demonstrated that increasing flavonoids concentration, naringenin, and quercetin, to the liquid artificial diet significantly increased the pre-reproductive period, decreased fecundity, and increased mortality of adult *apterae*. Addition of flavonoids to the diet also increased the average time of generation development and reduced the intrinsic rate of natural increase. Negative effects of flavonoids on herbivore performance (e.g., reduced growth, pupal mass, and fecundity, and increased mortality) have been previously proved (Alonso et al. 2002; Hare 2002a, b). Ruuhola et al. (2001) found that levels of the most abundant chemicals in the leaves of *Salix* spp., correlated markedly, but negatively with both consumption and growth. Lahtinen et al. (2004) showed that flavonoid aglycones reduced the growth rate and prolong the duration of the first instar *Epirrita autumnata* larvae. Contents of both total flavonoid and individually flavonoid compounds have been shown to reduce the larval performance of certain mid to late and late, sawfly species (Lahtinen et al. 2006) and one of the painted compounds (naringenin 4′,7-dimethylether) reduced the growth rate of late season species *Nipaecoccus viridis*. Birch leaf surface flavonoid aglycones have been shown to correlate negatively with the growth rate of the fifth instar and the pupal mass of the lepidopteran *Epirrita autumnata* (Valkama et al. 2005).

Flavonoids bioinsecticidal effect to insects might be explained especially by effects on insect feeding behavior. Goławska and Łukasik (2012) showed detrimental effects of the isoflavone genistein and the flavone luteolin on the feeding behavior of the pea aphid, *A. pisum*. Addition of genistein and luteolin to the sucrose–agarose gels generally prolonged the period of stylet activity, reduced salivation into the gel and passive ingestion of fluids from the gel and at higher concentrations (≥100 μg cm^−3^ for luteolin, ≥1,000 μg cm^−3^ for genistein), the flavonoids completely stopped these activities. Our study, which used EPG recordings demonstrated that pea aphid feeding behaviors on sucrose–agarose gels were clearly affected by the flavonoids naringenin and quercetin. The EPG data indicated that the flavonoids reduced aphid ingestion. Naringenin at 100 μg cm^−3^ reduced ingestion from fluids of the gels (g-E2 waveform) and at levels ≥1,000 μg cm^−3^ blocked ingestion (no g-E2 waveform was detected). Similar results were obtained for quercetin. Because the EPG waveform pattern g-E2 observed for aphids feeding on gels is similar to the waveform E2 observed when aphids ingest phloem sap on the plant (Tjallingii 1990), we infer that higher concentrations of tested flavonoids in phloem might reduce aphid activities associated with ingestion of phloem sap. The study presented here represents the first step in this area of research in this group of insects. Although, plants contain a great diversity of flavonoids (Harborne and Turner 1984) and the proportion studied in any form of biological system is low as yet it is difficult to predict whether they might or might not influence insect behavior (Simmonds 2001). It has been shown that flavonoid glycosides in alfalfa affect feeding behavior of pea aphid (Goławska et al. 2010, 2012). Morimoto et al. (2000) showed that flavonoids can act as feeding deterrents. They can act as endocrine disruptors in mammalian systems, having high binding affinities for estrogen receptors and have been shown to disrupt animal reproduction (Shutt 1976; Setchell et al. 1987). Recently, it has been also shown to bind the ecdysone receptor of insects (Oberdorster et al. 2001).

The results presented here suggest that the negative effects of plant flavonoids on the development of aphids could be a consequence of shortening or suppressing the feeding process. Furthermore, these data support the hypothesis that the mode of insecticidal activity of flavonoids is connected with their influence on the feeding behavior of insect (Simmonds 2001). A potential defense compound may act in three ways: it may decrease the consumption, it may reduce assimilation efficiencies and digestion, and it may act as a toxin (Scriber and Slansky 1981). In this study, the flavonoids naringenin and quercetin deterred aphid probing and feeding. The results confirm the reports by others, who suggested that flavonoids can have negative effects on herbivores (Simmonds and Stevenson 2001; Yu et al. 2003). Detailed investigation on the mechanisms by which flavonoids modulate behavior, especially feeding behavior, remains unknown (Simmonds 2003). Flavonoids and isoflavonoids are biosynthesized via the phenylpropanoid pathway (the key enzyme: phenylalanine ammonia-lyase, PAL) and contribute to plant defense against stressors (Dakora and Phillips 1996), such as pathogens, herbivores, or abiotic factors.

In summary, this work establishes detrimental effect of the flavanone naringenin and flavonol quercetin on the pea aphid, *A. pisum*. The insecticidal activity of these compounds on *A. pisum* may involve effects on pea aphid feeding behavior and development. So, these chemicals may have potential for pea aphid control and could be useful chemicals in biotechnological strategies creating transgenic plants. But if strategies based on the use of transgenic crops expressing specific flavonoids are to be adopted, more information on their precise modes of activity and biological/toxicological effects are needed.
